# Stakeholder mapping: advancing research on sexual and reproductive health policies and income protection for cisgender and transgender female sex workers in Buenos Aires, Argentina

**DOI:** 10.3389/fpubh.2025.1655388

**Published:** 2025-10-23

**Authors:** Estefania Panizoni, María Eugenia Esandi, Virginia Zalazar, Ines Aristegui, Agustina Argüello, Nadir Cardozo, Georgina Orellano, Marcela Romero, Mona Loutfy, Sharon Walmsley, Valeria Fink, Adriana Duran, Zulma Ortiz, MAS por Nosotras Study Group, Adriana Durán, Sharon Wamsley, Valeria Fink, Inés Aristegui, Marcela Romero Georgina Orellano, Eugenia Esandi, Virginia Zalazar, María Macarena Sandoval, Nadir Cardozo, Agustina Argüello, Mona Loufty, Silvina Vulcano, Carolina Pérez, Ana Gun, Emilia Frontini, Ana Zeltman, Camila Serrao, Romina Caballero, Estefania Panizoni, Maria Celia Trejo, Rodrigo Acuña, Mariana Duarte, Solange Fabian, Ariadna Exner, Susana Cahn, Debora Fiore, Florencia Gadea, Rocío Isaurralde, Valeria Álvarez, Emanuel Fojo, Julián Ylarri, Javier Mariani, Gissella Mernies, Mariela Ceschel, Agustín Nava, Oscar Cetrángolo, Ariela Goldsmith, Cecilia Valeriano, Amelia González, Adriana Corera, Gabriela Paz Raffo, Ana Inés Alvarez, Jonathan García, Daniela Parera, María Victoria Iannantuono, Julián García, Luciana Spadaccini, Herman Ludvik, Felipe Bilbao, Carina Cesar, Florencia Cahn, Carmen Ryan, Leandro Cahn, Mar Lucas, Pedro Cahn, Zulma Ortiz, María Inés Figueroa

**Affiliations:** ^1^Fundación Huésped, Research Department, Buenos Aires, Argentina; ^2^Asociación de Travestis, Transexuales y Transgenero de Argentina, Buenos Aires, Argentina; ^3^Sindicato de Trabajadoras Sexuales, Buenos Aires, Argentina; ^4^Women’s College Hospital, University of Toronto, Toronto, ON, Canada; ^5^University Health Network, University of Toronto, Toronto, ON, Canada; ^6^Coordinación Salud Sexual, VIH e ITS, Ministerio de Salud, Buenos Aires, Argentina

**Keywords:** stakeholder mapping, sexual and reproductive health, female sex workers, community-based participatory research, Argentina, transgender female sex workers, transgender and cisgender female sex workers

## Abstract

**Introduction:**

In the initial steps towards the development of an implementation project aimed to support sexual and reproductive health (SRH) policies and income protection for cisgender and transgender sex workers in the Ciudad Autónoma de Buenos Aires (CABA), we employed stakeholder mapping. This is a crucial tool in health policy and systems and research to identify, categorize, and characterize key stakeholders involved in policy planning and implementation.

**Methods:**

Prospective stakeholder mapping was conducted between February and September 2023 through a series of internal meetings and consultations with relevant community organizations to identify key stakeholders involved in SRH of female sex workers (FSWs) in CABA. The stakeholder mapping included three stages: 1. Identification and categorization of stakeholders using primary and secondary sources; 2. Analysis of stakeholder knowledge, level of agreement/interest, and level of influence/power; and 3. Characterization of stakeholder positioning. The absolute and relative frequencies of key stakeholders were estimated, and the average values of knowledge, power/influence, and interest/agreement were calculated for each category. The results were represented in a matrix identifying six types of positions (promoter, supporter, neutral, observer, high-risk blocker, low-risk blocker).

**Results:**

A total of 147 key actors were identified across sectors, including government, civil society, academia, abolitionist community organizations, health services, media and national and jurisdictional governments. Only four categories had detailed knowledge of the SRH situation and policies focused on FSWs. The stakeholders were categorized as 16% as promoters, 68% as supporters, 10% as blockers, 3% as observers, and 3% as neutral. Among promoters, national and jurisdictional governments stood out, while the supporters included the FSWs and the civil society organizations representing them, who also actively participated in the mapping process. Blockers mainly included abolitionist community organizations and security forces.

**Discussion:**

Stakeholder mapping proved to be a valuable tool for understanding the political landscape while ethically centering the voices of FSWs. The findings support the development of inclusive, context-sensitive policies and provide a replicable methodology for similar initiatives in other socio-political contexts.

## 1 Introduction

During the COVID-19 pandemic, female sex workers (FSWs) faced severe hardships and unprecedented marginalization, being unable to work and/or access essential health services and aid, especially in low- and middle-income countries (1, 2). In Argentina, FSWs remain among the most vulnerable and discriminated populations. Although sex work is not illegal in Argentina, it is a criminalized activity, and those who work on the streets are exposed to physical and sexual violence, including police harassment (3–6). These conditions were exacerbated during the pandemic, creating additional barriers to accessing sexual and reproductive health (SRH) services and social protection (7).

In 2022, under the *Women Rise* initiative promoted by the International Development Research Center (IDRC) (8), Fundación Huésped, and the Government of the Ciudad Autónoma de Buenos Aires (CABA), the action-research project “MAS por Nosotras” was initiated. This two-year project actively involved FSWs and their representative organizations -Asociación de Mujeres Meretrices de Argentina (AMMAR) and Asociación de Travestis Transexuales y Transgéneros de Argentina (ATTTA) -in seeking comprehensive solutions to the highest priority sexual and reproductive health (SRH) problems. Both the identification of this population’s health needs and the design of solutions require a transdisciplinary and multi-institutional approach. The inclusion of FSWs, plus the identification and engagement of the multiple actors involved in implementing policies and/or providing health services for this population strengthens this work by allowing identification of strains, enables discussion of conflicts of ideas and positions, and enables optimization of existing or potential synergies among these multiple actors. This in turn enables the design of strategies that balances agreements and disagreements to ensure the viability and sustainability of the proposed solutions.

Stakeholder mapping is a foundational tool in this context. Defined by Varvasovszky and Brugha (9), it refers to a set of methods to generate knowledge about individuals, groups, or organizations involved in planning or implementing an intervention—examining their behaviors, intentions, relationships, interests, influence, and power. Stakeholder mapping contributes to effective policy design by revealing the dynamics among key actors, clarifying how interventions operate within specific systems, and amplifying the perspectives of affected communities—particularly when those communities are directly engaged in the mapping process.

In *MAS por Nosotras*, the stakeholder mapping was conducted during the project’s initial phase to identify actors critical to the development and viability of interventions tailored to the SRH of FSWs in CABA. This article presents the main findings of the mapping and reflects on its contributions to strengthening context-responsive and community-informed implementation strategies. Specifically, it aimed to identify and categorize key actors connected to the SRH of FSWs in CABA and/or policies, plans, and programs focused on health and income protection for this group. The analysis includes their level of influence/power and interest/agreement, as well as an examination of their positions regarding the design and implementation of a comprehensive package of interventions that address their priority health needs and challenges.

## 2 Materials and methods

### 2.1 Research design

A prospective stakeholder mapping of key actors was conducted during the initial stage of executing the “MAS por Nosotras” project (February to September 2023) using the structured and iterative approach proposed by Hyder et al. (10). The results of identifying, categorizing, and analyzing the positions of key actors were reported using the Report on the Results of Key Actor Analysis (RISA tool) (11). RISA provides a structured framework to report the results of a stakeholder analysis. The items are structured in different domains (context, stakeholder identification, stakeholder interests (“stakes”), differentiation /categorization /prioritization, relationships, and implications for engagement), corresponding to the steps for stakeholder analysis by Reed et al. The completed RISA checklist is provided in the Supplementary material to enhance transparency and reproducibility.

The stakeholder mapping was led by members of Fundación Huésped (ZO, MEE, IA, VZ, EP and NC—the latter also a member of ATTTA), and data collection lasted 4 months. Information was drawn from: (i) project policy briefs and technical notes; (ii) stakeholder rosters/lists compiled during scoping; (iii) meeting minutes and workshop notes from sessions with research and community partners; and (iv) desk review of public materials (press releases, institutional websites, and social media posts). These sources informed stakeholder identification and the consensual scoring of attributes. This reinforces the rigor and traceability of the information used to construct the stakeholder database.

### 2.2 Setting

The study was conducted in CABA, one of the twenty-four federal entities and the capital of Argentina. The city is organized into 15 Communes, each constituting a decentralized unit of political and administrative management. The Communes have both exclusive competencies, those that fall solely under the jurisdiction of each Commune (i.e., community-led services, social welfare services) and shared competencies that involve joint responsibilities with the City Government (i.e., primary healthcare, social welfare services) (12). The Ministry of Health (MoH) has a dedicated area, the Coordination of Sexual Health, HIV, and Sexually Transmitted Infections (STIs), responsible for ensuring the sexual and reproductive rights of the population, as well as coordinating and executing programs related to HIV prevention and treatment. Refer to this link for a complete description: https://buenosaires.gob.ar/salud/coordinacion-salud-sexual-vih-infecciones-de-transmision-sexual (13).

### 2.3 Population

The study population consisted of key actors involved in the SRH of FSWs in CABA. Key actors were defined as individuals, groups, or organizations with an interest in the SRH of FSWs in the CABA and/or in policies, plans, and programs related to health and income protection for this group. These actors could potentially be affected by these policies (positively or negatively) and/or might have, power or influence over their planning and/or implementation. Although the scope was primarily jurisdictional, national-level actors with potential influence in designing policies for FSWs in CABA were also identified.

### 2.4 Stages of the stakeholder mapping

The stakeholder mapping encompassed the following three stages:

#### 2.4.1 Identification and categorization of key actors

This stage utilized primary and secondary sources. The primary sources included a series of three stakeholder mapping workshops, involving Fundación Huésped research and advocacy teams and representatives from AMMAR and ATTTA, to identify and refine an initial list of key actors engaged in SRH. This list was later expanded through consultations with three key representatives of other community organizations and secondary sources. These last included documentary reviews from media outlets, social media platforms such as Instagram, Twitter, and, to a lesser extent, Facebook, scientific journals, and searches on national and international websites. The search was conducted using keywords such as “sex work,” “sex workers,” “specific actor names,” “organization/institution names,” “trans-travesties employment quota,” “abolitionism,” “regulation,” “prostitution,” “prostitutes,” “trafficking,” “sexual exploitation,” “pimp” and “pimping” as well as Boolean operators. Based on prior knowledge of and/or information gathered from primary and secondary sources, the research team categorized the actors according to their institution and sector, grouping them into categories such as beneficiaries, national government, local government, civil society organizations (CSOs), health service providers, professional organizations, academia/science, media, abolitionist community organizations, and security forces (see Table 1). The scope of each actor’s actions was categorized as national, jurisdictional, or neighborhood/territorial. This information was organized into a database, where each actor and the selected variables for their characterization were recorded.

**Table 1:** Operational definition of key actors’ categories identified in the stakeholder mapping.

Sector	Key actors/institutions
National Government	Individuals and agencies belonging to the national government, including ministries, undersecretaries and health, social development, women, gender and diversity, migration, access to justice, and gender-based violence departments.
Local Government	Individuals and agencies belonging to provincial or Buenos Aires City jurisdictional governments, including health areas (hospital and community health services), social development, access to justice, legislature/legislators, law enforcement, women, gender and diversity departments, migration, housing, and ombudsman.
Civil Society Organizations (CSOs)	Non-profit civil society organizations focused on the common welfare and engaged in issues related to sex work.
Abolitionists Community Organizations	Community organizations advocating for the abolition of sex work.
Academy/ Science	Individuals from academic and/or scientific fields representing universities, research institutes, or independent researchers.
Media	Journalists covering issues related to sex work or abolitionism.
Health Service Providers	Health professionals that provide services to sex workers, including doctors, psychologists, social workers, gynecologists, and endocrinologists.
Beneficiaries	Women sex workers who are recipients of the comprehensive health package to be implemented.
Professional Organizations	Organizations whose common professional interests and activities relate to or could relate to sex work by women.
Security forces	Individuals or organizations belonging to national and local law enforcement agencies.

Source: Own elaboration.

#### 2.4.2 Analysis of knowledge, level of agreement/interest, and influence/power

As shown in Table 2, the stakeholder attributes were scored by consensus on a series of level scales.

**Table 2:** Scoring level scales used for stakeholder knowledge, interest/agreement, power/influence.

Attribute	Scale	Definition
Knowledge	0 to 3	0 = none; 1 = limited; 2 = general; 3 = detailed knowledge of FSW’s SRH situation and policies
Interest/agreement	−3 to +3	(0) neither in favor nor against; (+1) low interest/agreement in favor; (+2) moderate interest/agreement in favor; (+3) high interest/agreement in favor; (−1) low interest/agreement against; (−2) moderate interest/agreement against; (−3) high interest /agreement against the proposal
Power/Influence	0 to 3	0 = none; 1 = low; 2 = intermediate; 3 = high power and influence to shape policies and implementation

Source: Own elaboration adapted from Hyder et al. (10).

The level of *knowledge* each actor had regarding the state of SRH and the comprehensive health and protection policies for FSWs was assessed by the research and advocacy team using the following scale: (0) no knowledge of the SRH situation of FSWs or the policies, plans, and programs tailored to this population in CABA; (1) limited knowledge about the SRH situation and the policies, plans, and programs but unaware of their purpose; (2) general knowledge about the SRH situation and the policies, plans, and programs, including their purpose; (3) detailed knowledge of the SRH situation of FSWs and the policies, plans, and programs, including their purpose and components.

*Interest/agreement* was defined as the motivation and perception of each actor regarding the implementation of a comprehensive package of SRH interventions focused on FSWs, particularly how it impacts their organization **—**considering both risks and benefits. *Power/influence* refers to the actor’s capacity to affect the planning and implementation of the comprehensive package. Sources of power/influence included political authority, financial capacity, technical competence, and leadership. The research team assessed these attributes, along with the knowledge level of each actor, based on their prior understanding and the information gathered during stage 1. Regarding the level of interest/agreement, a scale from −3 to +3 was used: (0) neither in favor nor against; (+1) low interest/agreement in favor; (+2) moderate interest/agreement in favor; (+3) high interest/agreement in favor; (−1) low interest/agreement against; (−2) moderate interest/agreement against; (−3) high interest /agreement against the proposal. The level of influence/power was similarly classified on a scale from 0 to 3: (0) no power; (+1) low power; (+2) intermediate power; (+3) high power.

#### 2.4.3 Characterization of the positioning of key actors

The information gathered was organized into a database, enabling the characterization of each key actor’s positioning based on their level of interest/agreement and power/influence. This approach followed the categories and matrix proposed by Hyder et al. (10). Actors were categorized as *promoters* (high agreement; high power); *supporters* (high agreement; low power); *observers* (low agreement; low power); *neutrals* (high power; low or no agreement); *blockers* (negative interest). The blocker category was further subdivided into *high-risk blockers* (high power) and *low-risk blockers* (low power).

### 2.5 Data analysis

**Figure 1:**
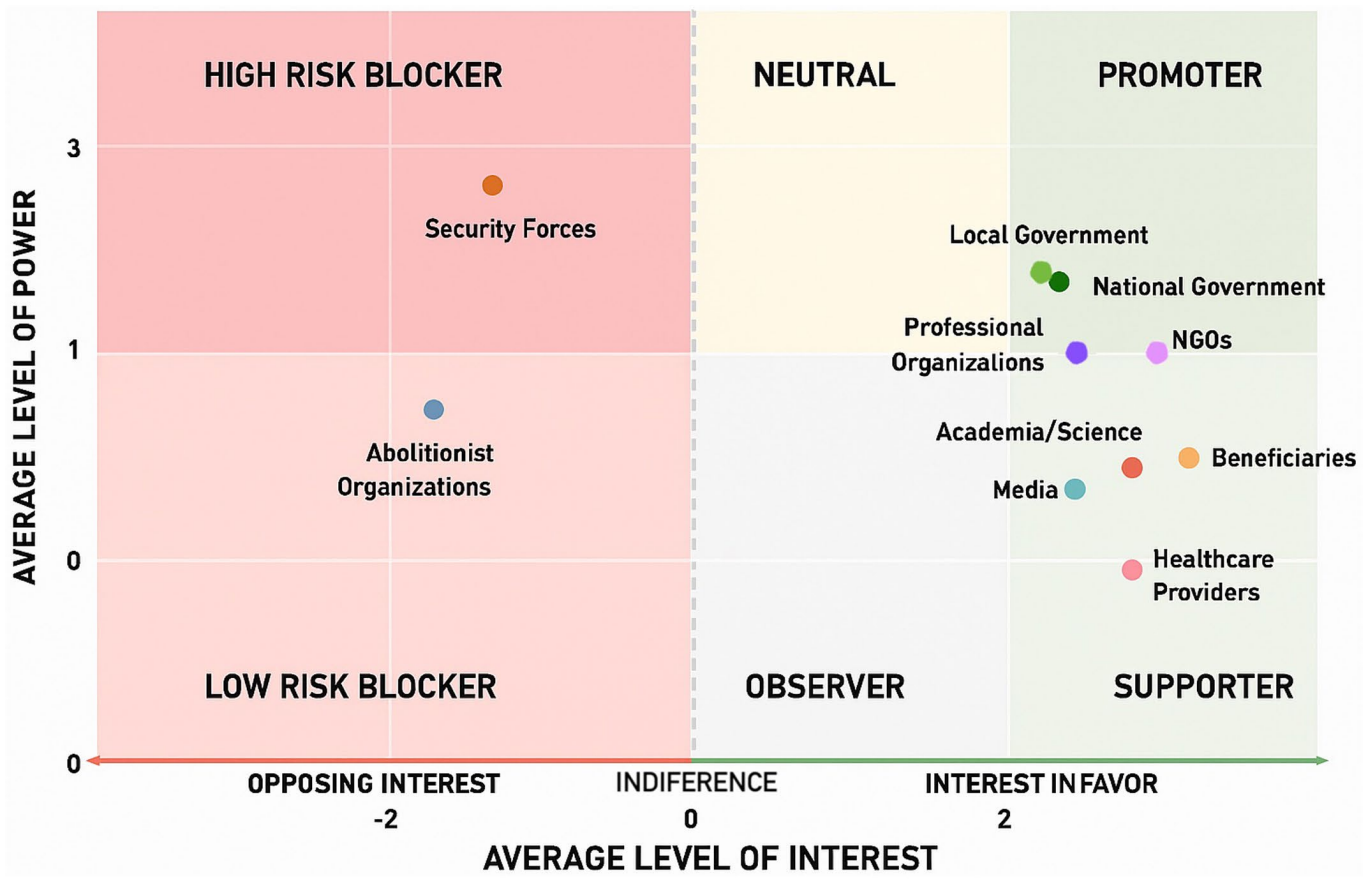
Positioning for each category of key actor according to their level of influence/power and agreement/interest in the formulation and implementation of the Comprehensive Package for FSW. Source: Own elaboration adapted from Hyder et al. (10).

Absolute and relative frequencies of key actors were estimated for each of the aforementioned categories, and the average values for knowledge, power/influence, and interest/agreement were calculated for each actor category. The results were visualized in a figure [adapted from Hyder et al. (10)], which identified the six described positions (see Figure 1). All analyses and the figure generated using R software.

### 2.6 Ethical aspects

The study protocol for the project “*MAS por Nosotras*” was approved by the Community Advisory Board and the Institutional Review Boards of Fundación Huésped to guarantee that the research envisages the safeguards provided by ethical and legal requirements established by national and international rules.

## 3 Results

The research team identified a total of 147 key actors. These actors represented governmental organizations, civil society organizations (CSOs), academic institutions, abolitionist community organizations (that oppose the legalization and/or regulation of sex work), national or local governments, as well as among health service providers and media representatives (Table 3).

**Table 3:** Key actors’ categories by sector.

Key actors’ categories	*N* (%)
Health service providers	40 (27%)
Local governments	33 (22%)
National government	21 (14%)
Civil Society Organizations	19 (13%)
Academy/Science	15 (10%)
Abolitionist Community Organizations	5 (3%)
Security forces^*^	5 (3%)
Media	3 (2%)
Professional organizations	3 (2%)
Beneficiaries	1 (1%)

* Security forces include national and jurisdictional forces.

Source: Own elaboration.

Almost one-third of the identified actors were *health care providers* involved in the care of FSWs. They included both Primary Health Care Centers located in neighborhoods where FSWs reside or work and specific services within certain municipal hospitals that provide care to these women, such as gynecology, infectious diseases, diversity, endocrinology, proctology, and psychology services. Generally, these actors are involved in the implementation of the comprehensive package of interventions developed under the “*MAS* por Nosotras” project.

The second most frequent category consisted of actors from *local government organizations*, representing 20% of the total. This category includes individuals working in certain Secretariats or Directorates of the government of CABA, whose responsibilities and functions are essential for the design and implementation of the comprehensive intervention package. It involves specific health-related agencies, such as the Coordination of Sexual Health, HIV, and Sexually Transmitted Infections (STIs), as well as others related to social determinants of health, such as the Institute of Housing, Social Inclusion, and Immediate Attention, the Ministry of Human Development and Habitat of CABA, among others.

Key actors from the *national government* represented the third most frequent category. This group included key actors working in national executive agencies involved in the formulation of policies related to health and/or social determinants affecting FSWs, such as the Ministry of Women, Gender, and Diversity, the Ministry of Health, specifically areas related to HIV, and the Ministry of Social Development. This group also included members of the legislative branch, specifically lawmakers who signed the bill on a comprehensive program for the promotion and protection of individuals in prostitution.

The fourth category, in terms of frequency, was represented by *civil society organizations* (CSOs) that advocate for the interests of FSWs (e.g., AMMAR and ATTTA) and others involved in different activities, such as non-formal education or anti-discrimination activism (e.g., “Positive Cycle”). In addition to these CSOs that protect the interests of FSWs, some community organizations with abolitionist positions were identified, opposing FSWs on ideological grounds.

*Academic actors* with extensive experience in research on women’s rights, diversity, and gender perspectives were also identified. *Security forces* at both national and jurisdictional levels were noted, with FSWs participating in the stakeholder mapping highlighting the significant impact of these actors on their work, due to traumatic and discriminatory situations and consequent effects on their mental health. Other identified actors included *professional associations and media representatives*. Finally, *FSWs* themselves were considered an essential key actor, as they are the target population and the ultimate beneficiaries of the intervention package.

The knowledge level across stakeholder categories varied, ranging from limited knowledge (i.e., media and security forces) to detailed knowledge (i.e., abolitionist community organizations, national government, CSOs, and beneficiaries).

Among the identified categories, 16% were characterized as promoters; 64% as supporters; 10% as blockers (5% high-risk and 5% low-risk), 3% as observers, and another 3% as neutral. Figure 1 illustrates the positioning of these categories, reflecting the average value of the two dimensions analyzed for each category (power/influence and agreement/interest).

The majority of promoters include actors from the national government (e.g., the Ministry of Women, Social Development) and the local government (e.g., Public Defender’s Office of CABA, the Ombudsman’s Office, or the HIV/STI Sexual Health Coordination of the MoH of CABA). Among the supporters are health providers, CSOs, professional organizations, academic/scientific institutions, media outlets, and the FSWs themselves.

Within the blockers, national and local security forces were categorized as high-risk due to their active opposition to the implementation of these policies. Most abolitionist community organizations related to sex work were also classified as blockers, although they posed a lower risk due to their limited power/influence in implementing policies focused on FSWs´ sexual and reproductive health.

When analyzing the power/influence and interest/agreement of the identified key actors, it becomes evident that, overall, they share similar positions.

## 4 Discussion

### 4.1 Key findings

This study is among the first to report both the methodology and results of a stakeholder mapping conducted as an initial step in a broader stakeholder engagement strategy within an implementation research project on the SRH of FSWs in Argentina.

A wide range of institutional actors—across government, health systems, civil society, academia and security forces—was found to play a role in shaping the policy landscape affecting FSWs. Health care providers were the most frequently identified stakeholder category, particularly those working in primary care centers and municipal hospitals that regularly serve FSWs. Several local and national government actors were also identified, underscoring the central role of public policies and institutional engagement in implementing the intervention package. Most actors were positioned as supporters with limited influence, while a smaller but highly influential group acted as promoters. Notably, community abolitionist organizations and security forces emerged as key blockers, reflecting both ideological and structural opposition to rights-based SRH policies for FSWs, highlighting potential barriers to implementation. Recognizing FSWs as both stakeholders and beneficiaries further reinforces the participatory features of the MAS por Nosotras project. These findings highlight the value of prospective stakeholder mapping in identifying political opportunities and risks early in the process of policy design and implementation. While other studies have characterized the attitudes of different actors towards sex work, few have included a diverse range of stakeholders from various sectors – such as government, health services, academia, media, FSWs, and their representing organizations- as comprehensively as this study (142). Additionally, this study provides a systematic assessment of their positions regarding the planning and implementation of health policies targeted at FSWs, considering their levels of interest/agreement and power/influence.

### 4.2 Policy implications

The stakeholder mapping provided crucial information for designing a strategic engagement plan that enhances the feasibility of implementing a comprehensive package of SRH interventions for FSWs in Buenos Aires (CABA). Promoters and supporters represent critical allies for policy advocacy and coalition-building, while blockers, such as security forces, represent major obstacles.

The findings revealed that, overall, there was a favorable scenario for promoting an SRH policy and comprehensive protection focused on FSWs in CABA, with a significant proportion of actors who would support or advocate for this intervention package. Notably, government officials at national and local levels stand out as supporters of designing targeted policies for FSWs. However, it is essential to consider that there was a change in authorities and the ruling party at the national level in December 2023, which have impacted the positioning of this group of actors. This shift may be less evident at the local level, where continuity of the ruling party is observed. These aspects highlight the temporality of stakeholder mapping related and the need for regular updates and triangulation with complementary data sources as highlighted by Varvasovszky and Brugha (9).

The mapping also helped identify barriers and potential sources of resistance. High-risk blockers, such as security forces, displayed both strong opposition and high levels of influence. Their stance was often underpinned by limited knowledge of FSWs’ realities and SRH issues, as well as a previously described conflict with FSWs. Conversely, abolitionist community organizations, while strongly opposed to the recognition of sex work as labor, had lower influence and were categorized as low-risk blockers. Accordingly, engagement with high-risk blockers should be context-dependent and multi-component (e.g., tailored awareness activities embedded in clear governance and accountability arrangements), whereas for low-risk blockers dialogue and selective coalition-building may be more feasible. These findings are consistent with international evidence on both the risks and opportunities of engaging security actors (14).

In the systematic review by Ma et al. (15), 49 studies were identified that explored the attitudes of different key actors to sexual activity and/or prostitution and found significant variability in the different positions, some of which even express a polarized position: on the one hand, they oppose prostitution, but on the other, they show empathy and understanding for the situation of FSWs and concern for their health and safety. Similar to our study, security forces were found to hold positions against prostitution/sex work, with limited knowledge of the activity. The review’s authors emphasized the importance of training these agents, as they interact significantly with these women during their work. Their stigmatizing and violent attitudes negatively impact FSWs’ health and self-care practices. Such situations were also reported by the women representing CSOs who took part in the stakeholder mapping conducted as part of the “*MAS por Nosotras*” project. The review also highlighted effective initiatives in India and Australia (16) that reduced stigmatizing behaviors by security forces. Effectiveness appears context-dependent and typically requires multi-component approaches -e.g., tailored awareness activities embedded within clear governance and accountability arrangements, alignment of incentives, and structured partnership with community organizations- rather than training alone. Consequently, engaging security actors in CABA should be pursued cautiously and as part of a broader, rights-based strategy across planning and implementation. This evidence would reinforce the need to involve and train these actors throughout the planning process of the SRH intervention package for FSWs in CABA.

Engagement with security actors in CABA should be cautious and rights-based, combining tailored awareness, governance/accountability, incentive alignment, and community partnership (not training alone) to mitigate harms driven by limited SRH knowledge yet high influence; with abolitionist organizations, emphasize respectful dialogue and persuasion, acknowledging ideological foundations and seeking limited, feasible cooperation. The stakeholder mapping process enabled a detailed characterization of the complex social system in which SRH and protection policies for FSWs are formulated and implemented. Using a methodology adapted from Schmeer (17) and refined by Hyder et al. (10) and Franco-Trigo et al. (11), the positions of stakeholders were visualized based on levels of interest, influence, and knowledge—offering a multidimensional understanding of the policy landscape. This approach contributed to the design of context-specific strategies aligned with the objectives of the *MAS por Nosotras* project, particularly its focus on developing sustainable responses that take into account existing power relations and institutional dynamics.

Finally, the stakeholder mapping process was grounded in Community-Based Participatory Research principles (18–21). FSWs and the organizations representing them were not only included as key stakeholders but were also actively involved in identifying and characterizing other actors. Their participation strengthened the legitimacy and relevance of the mapping results and reflected the values of citizen science by integrating the voices of those most directly affected. Moreover, their unique insights helped uncover actors and dynamics that might have remained invisible to external researchers or policymakers. This reinforces the notion that ethical policymaking in marginalized contexts must not only “include” but actively center the perspectives and lived experiences of affected communities.

### 4.3 Limitations

There are limitations in the study that must be acknowledged. First, the scope of the stakeholder mapping was jurisdictional, specific to CABA. While some national-level actors were included, other results might not capture provincial or regional dynamics, limiting generalizability to other contexts in Argentina and Latin America. Second, although RISA includes a section on relationships among stakeholders, our data sources did not provide sufficient, valid evidence to characterize inter-stakeholder ties. Future work should incorporate dedicated data collection (e.g., structured network instruments) to analyze relationships and their implications for implementation strategies. Third, the evaluation of each actor’s attributes relied on third-party assessments conducted by the research team. The absence of direct validation by stakeholders themselves introduces subjectivity. This evaluation may differ from the actors’ perspective, potentially influencing the matrix of agreement/interest and power/influence. Identifying and characterizing the actors relied on different sources of information and iteratively involved other individuals beyond the research and advocacy team. For instance, members of CSOs, with detailed knowledge of the challenges and current situation faced by FSWs, contributed significantly to this process. To minimize bias, characterization was made by consensus by the research team until agreement was reached. Third, although the mapping was conducted systematically and included actors from multiple sectors, it remains a snapshot of a dynamic sociopolitical landscape. Stakeholder positions and levels of influence can shift rapidly in response to political changes, institutional reforms, or shifting public discourses. This temporal limitation is inherent to stakeholder analysis, yet it underscores the importance of using such a tool as part of the process for implementation.

## 5 Conclusion

In conclusion, the stakeholder mapping conducted by the research and advocacy team and participating organizations provided valuable insights for the project. The results enabled a detailed understanding of the context in which policies concerning FSWs are designed and planned, informing strategies to engage and manage the participation of multiple actors. The involvement of FSWs in the process contributed a unique perspective, helping to reveal dynamics that might otherwise remain invisible to policymakers and researchers.

## Data Availability

The raw data supporting the conclusions of this article will be made available by the authors, without undue reservation.

## Group members of MAS por Nosotras Study Group

Adriana Durán, Sharon Wamsley, Valeria Fink, Inés Aristegui, Marcela Romero Georgina Orellano, Eugenia Esandi, Virginia Zalazar, María Macarena Sandoval, Nadir Cardozo, Agustina Argüello, Mona Loufty, Silvina Vulcano, Carolina Pérez, Ana Gun, Emilia Frontini, Ana Zeltman, Camila Serrao, Romina Caballero, Estefania Panizoni, Maria Celia Trejo, Rodrigo Acuña, Mariana Duarte, Solange Fabian, Ariadna Exner, Susana Cahn, Debora Fiore, Florencia Gadea, Rocío Isaurralde, Valeria Álvarez, Emanuel Fojo, Julián Ylarri, Javier Mariani, Gissella Mernies, Mariela Ceschel, Agustín Nava, Oscar Cetrángolo, Ariela Goldsmith, Cecilia Valeriano, Amelia González, Adriana Corera, Gabriela Paz Raffo, Ana Inés Alvarez, Jonathan García, Daniela Parera, María Victoria Iannantuono, Julián García, Luciana Spadaccini, Herman Ludvik, Felipe Bilbao, Carina Cesar, Florencia Cahn, Carmen Ryan, Leandro Cahn, Mar Lucas, Pedro Cahn, Zulma Ortiz, María Inés Figueroa.
