# Supplementary material for: Stakeholder mapping: advancing research on sexual and reproductive health policies and income protection for cisgender and transgender female sex workers in Buenos Aires, Argentina
Source: Front Public Health. 2025 Oct 23;13:1655388. doi: 10.3389/fpubh.2025.1655388 (PMC12588998; doi:10.3389/fpubh.2025.1655388)
Supplement: Supplementary file 1 [file Data_Sheet_1.docx]

**Supplementary Material**

**RISA Template (Reporting Items for Stakeholder Analysis)**

Use this template to systematically report a stakeholder analysis according to the RISA tool. Complete each section and, where applicable, use the “Page/Section” and “Reason for non-applicability” columns

1. **Context**

| **Reporting item** | **Clarifications/Guide** | **Page/Lines (Section)** | **Reason for non-applicability** |
| --- | --- | --- | --- |
| **Objective of the stakeholder analysis** | What were the specific objectives of the analysis? | P4, lines 84-86 (Introduction) |  |
| **Reasons for conducting the analysis** | How was the analysis expected to support planning? | P4, lines 82-83 (Introduction) |  |
| **Scope or level of the analysis** | Local, regional, state/national, or international? | P4, lines 84-86 (Introduction) P5, lines 93-101 (research Design) |  |
| **People who conducted the analysis** | Who identified, classified, and analysed? Indicate roles. | P5, lines 102-109 (Research design) |  |
| **Duration of data collection** | How long did data collection take? | P4, lines 103 (Research Design |  |

1. **Application of stakeholder analysis methods**

**2a) Stakeholder identification**

| **Reporting item** | **Clarifications/Guide** | **Page/Section** | **Reason for non-applicability** |
| --- | --- | --- | --- |
| **Definition of ‘stakeholder’ used** | Operational definition applied to decide who is a stakeholder | P5, lines 123-128 (Population) |  |
| **Steps/Process followed** | Sequence of steps to identify stakeholders | P6, lines 130-182 (Stages of stakeholder mapping) |  |
| **Information sources** | Who provided the information or where was it obtained from? | P6, lines 130-177 (Stages of stakeholder mapping) |  |
| **Data collection methods** | e.g., literature review, interviews, workshops | P6, lines 102-109,130-177 (Stages of stakeholder mapping) |  |
| **Presentation of results** | How are findings displayed? e.g., narrative, tables, maps | P 8-10, lines 188-241 P14-17,367-384 (Tables and figure) |  |

**2b) Identification of stakeholder interests/‘stakes’**

| **Reporting item** | **Clarifications/Guide** | **Page/Section** | **Reason for non-applicability** |
| --- | --- | --- | --- |
| **Steps/Process followed** | Sequence of steps to identify the ‘stakes’ | P6-7, lines 149-176 (Stages of stakeholder mapping) |  |
| **Information sources** | Who provided the information or where was it obtained from? | P7, lines 163-165 (Stages of stakeholder mapping) |  |
| **Data collection methods** | e.g., literature review, interviews, surveys. | P6, lines 132-141 (Stages of stakeholder mapping) |  |
| **Data analysis** | Techniques used to analyse the ‘stakes’. | P7, lines 177-188 (Stages of stakeholder mapping) |  |
| **Presentation of results** | How are findings presented? e.g., tables, figures | P17 (figure), lines 382-384 |  |

**2c) Differentiation/Categorisation/Prioritisation of stakeholders**

| **Reporting item** | **Clarifications/Guide** | **Page/Section** | **Reason for non-applicability** |
| --- | --- | --- | --- |
| **Attributes and their definition** | e.g., power, interest, legitimacy; define each attribute | P7, lines l63-169 (Stages of stakeholder mapping) |  |
| **Steps/Process followed** | Sequence to differentiate/categorise/prioritise. | P6, lines 149-150 (Stages of stakeholder mapping) |  |
| **Information sources** | Who/what sources support the attributes and their scoring? | See 2a/2b |  |
| **Data collection methods** | e.g., interviews, matrices, workshops | See 2a/2b |  |
| **Data analysis** | Analytical techniques for attributes/categories | P7, lines 178-182 (Stages of stakeholder mapping) |  |
| **Presentation of results** | e.g., narratives, tables, matrices | P14-17, lines 367-384 (Tables and figure) |  |

**2d) Investigation of relationships among stakeholders***

| **Reporting item** | **Clarifications/Guide** | **Page/Section** | **Reason for non-applicability** |
| --- | --- | --- | --- |
| **Steps/Process followed** | Sequence of steps to identify relationships |  |  |
| **Relationships analysed** | e.g., alliances, communication, collaboration |  |  |
| **Time frame of the relationships** | e.g., past year, current, potential; willingness to collaborate. |  |  |
| **Information sources** | Who/what sources provided relationship data? |  |  |
| **Data collection methods** | e.g., interviews, surveys, document review. |  |  |
| **Data analysis** | e.g., SNA metrics (density, centrality) or other approaches |  |  |
| **Presentation of results** | e.g., narratives, sociograms, maps |  |  |

(*) Not reported. Methods used do not allow a proper characterization of relationships among stakeholders

1. **Future actions and stakeholder engagement**

| **Reporting item** | **Clarifications/Guide** | **Page/Section** | **Reason for non-applicability** |
| --- | --- | --- | --- |
| **Use of results and engagement strategies** | How will the results be used? What engagement strategies derive from the findings? How did the findings influence stakeholder involvement? Recommendations | P11-12/Policy Implications |  |

Note: This template is based on the RISA tool (Reporting Items for Stakeholder Analysis) developed from Franco-Trigo L. et al., Health Policy 2020, and the author’s PhD thesis (UTS).
